# Sex hormone-binding globulin controls sex-specific lipolytic activity in human abdominal subcutaneous adipocytes

**DOI:** 10.1016/j.molmet.2025.102189

**Published:** 2025-06-16

**Authors:** Julie Abildgaard, Aiste Aleliunaite, Carla Horvath, Nagendra Palani, Tora Ida Henriksen, Jiawei Zhong, Katja Munch Lorentsen, Victor Svenstrup, Hanne Frederiksen, Anders Juul, Camilla Charlotte Scheele, Søren Nielsen

**Affiliations:** 1 The Centre for Physical Activity Research, Rigshospitalet, University of Copenhagen, Copenhagen, Denmark; 2 Novo Nordisk Foundation Center for Basic Metabolic Research, University of Copenhagen, Copenhagen, Denmark; 3 Department of Growth and Reproduction, Copenhagen University Hospital Rigshospitalet, Copenhagen, Denmark; 4 International Center for Research and Research Training in Endocrine Disruption of Male Reproduction and Child Health (EDMaRC), Copenhagen University Hospital Rigshospitalet, Copenhagen, Denmark; 5 Department of Clinical Medicine, University of Copenhagen, Copenhagen, Denmark; 6 Department of Medicine Huddinge (H7), Karolinska Institutet, Karolinska University Hospital Huddinge, SE-141 83, Huddinge, Sweden

**Keywords:** Sex-hormone binding globulin, Adipocyte, Lipolysis

## Abstract

Regulation of lipid metabolism is fundamental for metabolic health, and adipose tissue is a central component in this process. Adipose tissue differs considerably between women and men in terms of a higher subcutaneous capacity for storage, which is linked to metabolic health, in women. Sex hormone-binding globulin (SHBG) contributes to the regulation of circulating sex hormone bioavailability and has been shown to predict risk of metabolic dysfunction. Here, we investigate the sex-specific relationship of SHBG with metabolic status and adipocyte-dependent lipolysis. We measured serum concentrations of sex hormones, SHBG, fasting glucose, and insulin in a cohort of 63 women and 27 men from which adipose biopsies were collected and mature adipocytes isolated. In women, high serum SHBG concentrations were strongly associated with low *in vivo* Homeostatic Model Assessment for Insulin Resistance (HOMA-IR), and lower unstimulated *ex vivo* lipolysis but higher isoprenaline stimulated *ex vivo* lipolysis. In contrast, no effect of SHBG on the above-mentioned parameters were observed in men. In vitro cultured human adipocytes also increased lipolytic activity in response to SHBG, but only in the absence of testosterone, suggesting that testosterone inhibits the catecholamine-induced lipolysis of SHBG in adipose tissue. In conclusion, we identify SHBG as a novel sex-specific regulator of adipocyte lipolysis and lipid metabolism. At the same time, our data emphasize sex-dependent effects of SHBG on adipocyte lipid metabolism, and we propose testosterone binding to SHBG as a driving factor mediating these sex differences.

## Highlights

• SHBG is linked to lower HOMA-IR and altered lipolysis in women, but not men.
• High SHBG reduces basal lipolysis but enhances stimulated lipolysis in women.
• SHBG increases lipolysis in adipocytes, but only without testosterone.
• Testosterone inhibits SHBG-driven catecholamine-induced lipolysis in adipose tissue.
• SHBG is internalized in adipocytes.

## Graphical abstract

**Figure:**
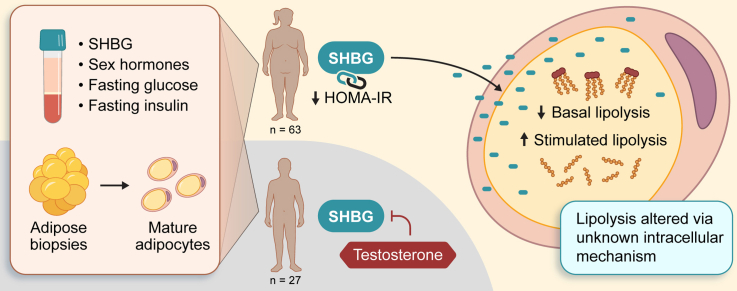

## 1 Introduction

Sex hormones play an important role in regulating adipose tissue distribution and adipocyte metabolism [[1], [2], [3]]. In the circulation, sex hormone bioavailability is mainly regulated by protein binding to sex hormone-binding globulin (SHBG) and albumin, leaving only a small fraction of sex hormones unbound and able to exert its biological effects on peripheral tissues [4,5]. Studies further indicate an independent role of SHBG in obesity and metabolic diseases, but the mechanisms remain largely unresolved. Moreover, it is widely discussed whether SHBG should only be used as a biomarker of systemic metabolic risk status or whether it has hepatokine-like independent metabolic effects [6]. In relation to this, several studies have shown that low serum SHBG in overweight individuals is associated with an increased risk of metabolic syndrome, type 2 diabetes, and cardiovascular disease [[7], [8], [9], [10]]. At the same time, GWAS show that specific polymorphisms in the SHBG gene, associated with low concentrations of circulating SHBG, predict an increased risk of lipid metabolic diseases and type 2 diabetes, partly through increased ectopic lipid deposition, indicating that SHBG *per se* exerts systemic metabolic effects [[11], [12], [13], [14]]. Interestingly, some observational studies find that the protective effects of high circulating SHBG on the risk of ectopic lipid deposition and type 2 diabetes are more pronounced in women compared to men [14,15] while other studies indicate health related associations with SHBG in both sexes [[11], [12], [13]].

Previous mechanistic studies have highlighted the role of SHBG in modulating inflammation and lipid metabolism in rodents [16,17]. For instance, in a transgenic mouse model, overexpression of human SHBG protected against high-fat diet induced obesity and insulin resistance, mediated through induction of lipolysis in white adipose tissue, suggesting that this liver-derived factor can directly influence adipose tissue through a previously unexplored liver-to-adipose tissue signaling axis [16]. Furthermore, *in vitro* studies show an upregulation of proteins regulating lipolysis following SHBG treatment [16,17]. However, the potential sex-specific actions of SHBG on adipocytes remain unknown. Furthermore, unlike humans, adult rodents have no hepatic SHBG production, making previous findings difficult to translate into human relevance [18].

Several attempts have been made to identify an SHBG receptor and both G-protein coupled receptors and the endocytic receptor Megalin (also known as Low density lipoprotein receptor-related protein 2) have been suggested to mediate SHBG signaling [19,20]. Furthermore, it has been speculated that receptor binding of SHBG is affected particularly by the presence of testosterone, which binds with high affinity to SHBG, causing conformational changes in the protein [21].

In this study, we found a strong relationship between circulating SHBG concentrations and lipolytic function of subcutaneous adipocytes from overweight women but not men. Lipolytic activity of the adipocyte has been linked to body weight regulation in women where high basal lipolysis and low catecholamine stimulated lipolysis precede weight gain and insulin resistance [22]. These findings prone us to investigate whether SHBG can signal directly to the adipocyte and thereby regulate adipose tissue lipolytic activity and thus, represents the missing link between high circulating SHBG concentrations and protection against metabolic disease.

## 2 Methods

### 2.1 Human cohorts

Analyses were performed in samples from women (n = 63) and men (n = 27), recruited from two different cohorts. Sex was defined by self-reporting. The women were included from a longitudinal study investigating body weight development over time (NCT02227043) and has been described in detail before [22]. Briefly, overweight women of all ages were recruited to this study based on previous participation in studies related to adipose tissue metabolism performed in the lab. Three were postmenopausal and none were using oral estrogen preparations. The main purpose of the study was to investigate the influence of fat cell size/number and adipose function on weight development over very long time periods The men were included from a study investigating differences in adipose biology between non-obese individuals living with (n = 14, stimulated with oral antidiabetic agents) or without (n = 13) type 2 diabetes and has been described before [23]. All examinations in both studies were conducted in the morning after an overnight fast and persons with diabetes abstained from glucose lowering medications on the morning of investigations. Both studies were approved by the Ethics Board of Stockholm and informed written consent was obtained from all participants.

### 2.2 Mature adipocyte isolation and assessment of lipolysis

Subcutaneous abdominal white adipose tissue biopsies were obtained by needle aspiration under local anesthesia. Mature adipocytes were isolated from the tissue pieces by collagenase treatment and mean fat cell size was determined as described previously [24]. Diluted fat cell suspensions (2%, vol/vol) were incubated for 2 h at 37 °C in an albumin/glucose-containing buffer (pH 7.4) [25] without (basal) or with increasing concentrations of the beta adrenoceptor-selective agonist isoprenaline. Glycerol release was determined using a luminometric assay [26]. Values at maximum effective concentration were used. Results were normalized to grams of lipid assessed through triglyceride extraction from adipocytes using heptane.

### 2.3 Measurements of sex hormones

All hormone analyses were performed at the Department of Growth and Reproduction, Rigshospitalet, Copenhagen, Denmark. Analyses of estradiol (E_2_) and testosterone in human serum were performed by isotope dilution online TurboFlow-LC-MS/MS as described previously [27,28]. LOD for E_2_ was 4.04 pmol/L and interassay coefficient of variation (CV) 5.7 %. LOD for testosterone was 0.12 nmol/L and CV was 1.8 %. Calculation of free E_2_ was done based on Mazer [29]. Calculation of free testosterone was performed using the Vermeulen formula [30]. SHBG concentration was determined by a chemiluminescence immunoassay (Access2, Beckman Coulter, Brea, CA, USA) with a LOD of 0.33 nmol/L and interassay CV of the SHBG assay of <9%.

### 2.4 Human preadipocytes

Human adipogenic progenitor cells were isolated from the stromal vascular fraction of the biopsies on the day they were obtained from abdominal subcutaneous adipose tissue from fertile women. Isolated cells were expanded and frozen in liquid nitrogen in a proliferative state as previously described until the onset of the study [31]. All subjects provided written consent and the studies were performed in accordance with the declaration of Helsinki. The cell studies were approved by the Danish Data protection agency, Denmark, RH-2017-60 I-suite nr: 05329.

### 2.5 Cell culturing

Isolation and culturing of human adipocyte progenitors are described in detail elsewhere [31]. Briefly, biopsies were collected in DMEM/F12 (Gibco) with 1% penicillin/streptomycin (Life Technologies) and were digested with 10 mg collagenase II (C6885-1G, Sigma) and 100 mg BSA (A8806-5G, Sigma) in 10 ml DMEM/F12 for 20 min at 37 °C while gently shaken. The suspension was then filtered, washed with DMEM/F12, resuspended in DMEM/F12, 1% PS, 10% fetal bovine serum (FBS) (Life Technologies), and seeded in a 25 cm^2^ culture flask. Media was changed the day following isolation and then every second day until cells were 80% confluent after which they were split into a 10 cm dish (passage 0). When cells were seeded for experiments, FGF-1 (1 nM; 11343557, Immunotools) was added to the media until full confluence was reached. Cells were expanded at 37 °C in an atmosphere of 5% CO_2_ and the media was changed every second day. Adipocyte differentiation was induced two days after adipocyte progenitor cultures were 100% confluent by removal of FGF-1 from the media and addition of a differentiation cocktail consisting of DMEM/F12 containing 1% PS, 0.1 μM dexamethasone (Sigma–Aldrich), 100 nM insulin (Actrapid, Novo Nordisk or Humulin, Eli Lilly), 200 nM rosiglitazone (Sigma–Aldrich), 540 μM isobutylmethylxanthine (IBMX, Sigma–Aldrich), 2 nM T3 (Sigma–Aldrich) and 10 μg/ml transferrin (Sigma–Aldrich). After three days of differentiation, IBMX was removed from the cell culture media and cells were differentiated for an additional three days with the remaining differentiation compounds. Following this, rosiglitazone was removed from the media and cells were differentiated for an additional six days before the cells were considered fully differentiated. Long-term stimulation of adipocytes with purified human SHBG (Bio-Rad, Hercules, California, USA, Cat# PHP147) was done by adding SHBG to the media once, in relation to the last media change during differentiation (three days), in a concentration of 100 nM. Short-term stimulation of adipocytes with purified human SHBG was done by adding SHBG to the media for the last 2 h prior to experiments.

### 2.6 Assessment of lipolysis in cultured cells

Cells were incubated in Krebs–Ringer HEPES buffer containing 3.5 % Bovine Serum Albumin (Free fatty Acid Free) and 6 mM glucose, with/without 1 μM nor adrenaline (NE). After 2 h of incubation, the media was removed and stored at −80 °C until further analysis. 10 μl media was used for the free fatty acid quantification using a NEFA assay kit (FUJIFILM Wako, Richmond, VA, USA), an *in vitro* enzymatic colorimetric method assay for quantitative determination of non-esterified fatty acids in cultured media. The assay was performed in NUNC F96 immunoplates and data collected in a Sunrise Plate reader at 550 nm. In lipolysis experiments where adipocytes were co-stimulated with SHBG and sex hormones, SHBG, and/or testosterone (testosterone, catalog# 16441, Thermo Fisher Scientific, diluted in 99 % ethanol) or estradiol (β-estradiol, catalog#L03801-14, Thermo Fisher Scientific, diluted in 99% ethanol) was added to the media in increasing concentrations (SHBG: 0 nM, 10 nM, 50 nM, 100 nM, estradiol: 0 nM, 1 nM, 5 nM, 10 nM, and testosterone: 0 nM, 5 nM, 25 nM, 50 nM) the last three days of differentiation from where lipolysis experiments were performed as described above.

### 2.7 Oxygen consumption in cultured adipocytes

Oxygen consumption was measured using a Seahorse Bioscience XF96 Extracellular Flux Analyzer (Agilent Technologies, Santa Clara, California) according to the manufacturer's protocol. Preadipocytes were expanded until reaching 100% confluency and were then seeded in Seahorse plates and differentiated as described above. Experiments were performed on day 12 of differentiation and human purified SHBG was added to the media at a concentration of 100 nM. The results were extracted from the Seahorse Program Wave 2.2.0. Baseline measurements of OCR were performed for 33 min before NE or saline was added and measurements of the concomitant responses were recorded for 65 min. All other states were induced using the Seahorse XF Cell Mito Stress test kit according to the manufacturer's protocol. After 98 min, leak state was induced by adding Oligomycin, which inhibits the ATP synthase. The leak state measurements were recorded for 20 min, after which the ionophore (carbonyl cyanide-4-(trifluoromethoxy) phenylhydrazone) (FCCP) was added, which collapses the proton gradient across the mitochondrial inner membrane resulting in a completely uncoupled state. After an additional 30 min Antimycin A and Rotenone were added to inhibit complexes III and I respectively, resulting in only non-mitochondrial respiration. These measurements were recorded for 20 min.

### 2.8 Bulk RNA-sequencing of cultured adipocytes

Adipocytes were stimulated with 100 nM SHBG in regular differentiation media for the last three days of differentiation. RNA (1000 ng) was extracted from adipocytes using the Trizol method. Bulk RNA-seq data was generated through BGI Genomics (Denmark) in paired-end format. The adapter-trimmed FASTQs received from BGI were aligned to the GRCh38.p13 genome and the counts file generated using STAR aligner (v2.7.10a) [32]. The data was then analysed for differential expression using DESeq2 [33] in the R language (v4.1.0), with the design formula modeling donor of origin and SHBG treatment. Ensembl 105 [34] was used for the annotations. Gene ontology analysis was performed with g:Profiler [35] throught the gprofiler2 R package. No new methods were developed for analyses in this manuscript. RNA-seq data is publicly available on NCBI GEO datasets through the accession: GSE288362.

### 2.9 Blocking of endocytosis

For non-specific inhibition of endocytosis, 2-Deoxy-d-glucose (2-DG; Cat. No. 111980010, Thermo Fisher Scientific) was added to the cell culture medium on day 12 at a concentration of 50 nM for 30 min. This treatment was performed 30 min prior to a 30-minute incubation with SHBG.

To specifically silence key endocytic pathways, adipocytes were transfected with siRNA pools (ON-TARGETplus, Dharmacon) targeting DNM2 (Cat. No. L-004007-00-0005), LRP2 (Cat. No. L-012673-00-0005), and CAV1 (Cat. No. L-003467-00-0005). Transfections were carried out on day 6 of differentiation using 3 μl/mL of Lipofectamine® RNAiMAX Transfection Reagent (Thermo Fisher Scientific) and 20 nM siRNA in antibiotic-free Opti-MEM®/DMEM:F12 medium for 24 h. The cells were then fixated and stained as described in the Immunoflourescence section.

### 2.10 Immunoflourescence

Following a 30-minute SHBG (100 nM) stimulation cells were washed in 37 °C phosphate buffered saline (PBS) and fixed with neutral buffered formalin (10 %) for 15 min. Timing of SHBG stimulation was based on previous findings in lymphocytes [36]. Following fixation, cells were washed three times in Dulbecco's PBS (DPBS) (each wash 2 min) and permeabilized in TritonX-100 (0.1 %), washed again and blocked in 3 % bovine serum albumin (BSA) for 60 min. Primary antibody (Ab) (SHBG monoclonal Ab, Thermo Scientific Cat# MA526324) was dissolved 1:100 in Hank's balanced salt solution (HBSS) and cells were incubated for 60 min at 37 °C. Cells were then washed three times in DPBS and secondary Ab (Goat Anti-Mouse AlexaFlour 594, Thermo Scientific Cat# A-11005) was added for 20 min. The cells were once again washed three times in PBS and left in PBS through imaging using an EVOS M5000 microscope (ThermoFisher Scientific, Waltham, Massachusetts, U.S.).

### 2.11 Spatial transcriptomics

Spatial transcriptomics was performed using Visium Spatial Gene Expression (10x Genomics) on subcutaneous abdominal adipose tissue. The full spatial transcriptomics dataset has been published previously and the methods described in detail elsewhere [37]. Briefly, all Visium libraries were sequenced simultaneously on the Illumina NovaSeq6000 platform, at a sequencing depth of approximately 80–130 M read-pairs per sample. In accordance with the Visium protocol, the number of bases sequenced were 28 nt for R1, 120 nt for R2, and 10 nt for each of the indexes.

### 2.12 Single nuclei RNA (snRNA) sequencing

SnRNA sequencing analyses were performed based on the single cell and spatial transcriptomic map of human white adipose tissue published previously [38].

### 2.13 Statistical analyses

A power calculation, on the human cohorts, based on previously detected clinically significantly meaningful differences in stimulated adipose lipolysis, was performed [22]. We had 80% power to detect a 20% difference in stimulated lipolysis at a p value of <0.05 with 26 individuals included in each cohort. No previous studies investigated associations between SHBG and human adipose lipolysis. Thus, such data could not form the basis of the power calculation performed.

Data were log10-transformed when not normally distributed. Histograms and Q–Q plots were used to assess normal distribution (data not shown). Differences in variables between groups were compared using an independent samples t-test for continuous variables. Analyses of the association between sex hormone concentrations and adipocyte lipolysis were performed using a linear regression model. Models were checked for assumptions of the linear model, including normal distribution of the residuals, homogeneity of variance, linearity, and independent observations. As sensitivity analyses, linear regressions were investigated for potential outliers using the ROUT method with a Q-value of 1. Data with repeated measures (SHBG concentrations, testosterone concentrations) were handled using a mixed model for the outcome variable (lipolysis) and time adjusted for the interaction between the two covariates. A random effect accounting for an individual variation was included. A Tukey post-hoc test was performed to assess between and within group differences. Mixed model analyses were performed in SAS Enterprise Guide version 7.1. Other statistical analyses were performed using IBM SPSS statistics version 25. Statistical analyses on spatial transcriptomics and snRNAseq datasets are as described previously [37,38].

## 3 Results

### 3.1 Serum SHBG concentrations are associated with lipolytic function of mature subcutaneous adipocytes in women

To explore if serum SHBG concentrations were related to subcutaneous adipocyte metabolic function, we investigated the association between serum SHBG and *ex vivo* lipolytic capacity of the adipocytes in a cohort of 27 men and 63 women (*Subject Characteristics are shown in* Table 1*).* In the cohort of women, serum SHBG concentrations explained 17 % (p = 0.001) of the variation in basal lipolysis of mature abdominal subcutaneous adipocytes with high SHBG being associated with low basal lipolysis (Figure 1A). There were no associations between circulating SHBG and basal lipolysis in the cohort of men (p = 0.40) (Fig 1B). Similarly, circulating SHBG concentrations explained 17 % (p < 0.001) of variation in isoprenaline stimulated lipolysis in women with high SHBG being associated with high stimulated lipolysis (Fig 1C) and no association between SHBG and stimulated lipolysis in men (p = 0.52) (Fig. 1D). Interaction analyses for sex∗SHBG revealed significant sex dependent differences in the association between circulating SHBG and both basal lipolysis (p = 0.02) and isoprenaline stimulated lipolysis (p = 0.03). Importantly, correcting associations between SHBG and unstimulated and stimulated lipolysis for E_2_ or testosterone did not impact our findings indicating that SHBG could have sex hormone independent effects on lipolysis in adipocytes (Supplemental Fig. 1a–h and Supplemental Table 1). In women, serum SHBG was further responsible for 19 % (p < 0.001) of the variation in insulin sensitivity, with low SHBG being associated with insulin resistance (Fig 1E). Statistical mediation revealed that basal lipolysis in the mature adipocytes explained 24 % (95 % CI: 1–47%) and stimulated lipolysis explained 28 % (95 % CI: 5–52 %) of the association between serum SHBG and insulin sensitivity. In men, no associations were found between serum SHBG and insulin sensitivity (p = 0.61) (Fig 1F).

**Table 1:** Subject Characteristics.

	Men	Women
N	27	63
Age, years	63 (51–68)	39 (32–42)
BMI, kg/m^2^	26.2 (25.1–27.6)	35.0 (32.8–39.4)
HOMA-IR, AU	1.8 (1.4–3.3)	2.6 (1.8–3.8)
SHBG, nmol/L	32.9 (25.5–56.3)	34.6 (27.3–48.3)
LH, IU/L	3.9 (2.4–5.3)	3.5 (1.7–7.9)
FSH, IU/L	–	5.1 (3.5–6.5)
E_2_, pmol/L	85 (67–102)	183 (109–325)
Free E_2_, pmol/L	1.6 (1.4–2.1)	3.5 (2.0–6.9)
T, nmol/L	13.9 (12.4–18.1)	–
FT, pmol/L	284 (243–344)	- -
Postmenopausal, n		3

Data are presented as median (interquartile range).

LH, Luteinizing hormone. FSH, follicle stimulating hormone. E_2_, estradiol. T, testosterone. FT, Free Testosterone.

**Figure 1:**
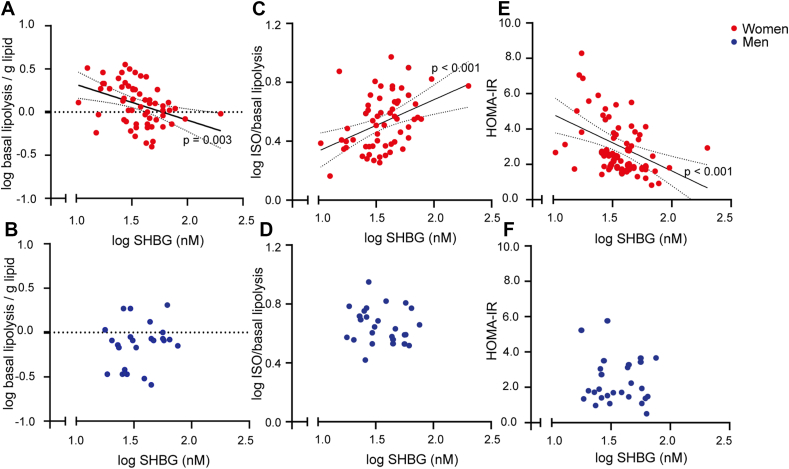
**Associations between *ex vivo adipocyte lipolysis and serum sex hormone-binding globulin (SHBG) in men and women****.* ***a)*** Serum SHBG and basal lipolysis in women (N = 67). ***b)*** Serum SHBG and basal lipolysis in men (N = 23). ***c)*** Serum SHBG and isoprenaline stimulated lipolysis in women (N = 67). ***d)*** Serum SHBG and isoprenaline stimulated lipolysis in men (N = 23). ***e)*** Serum SHBG and HOMA-insulin resistance index (HOMA-IR) in women (N = 67). ***f)*** Serum SHBG and HOMA-IR in men (N = 23).

### 3.2 *In vitro* SHBG stimulation controls lipolytic activity and lipid metabolic processes in cultured adipocytes

Based on these findings, we hypothesized that the observed lipolytic regulation is directly dependent on SHBG, while being inhibited when sex hormones are bound to SHBG. We assessed this hypothesis in *in vitro* models of human subcutaneous adipocytes. To test the effect of SHBG on adipocytes in a sex-hormone free environment, we stimulated cultured human subcutaneous adipocytes, harvested from fertile women, with human purified SHBG acutely and long-term, *in vitro*. While SHBG stimulation led to a decrease in basal lipolysis, NE stimulated lipolysis increased significantly under the presence of SHBG (SHBG × NE interaction, p = 0.001) (Figure 2A). Long-term SHBG stimulation had no effect on adipocyte density *in vitro* (Supplemental Fig. 2a). Furthermore, the maximal stimulated oxygen consumption rate (OCR) was increased in the adipocytes following short-term SHBG stimulation of both white (p = 0.02, Fig 2B) and brown (p = 0.01, Fig 2C) adipocytes. Long-term stimulation with SHBG did not influence OCR, as assessed in a model of human thermogenic adipocytes used to achieve a maximal OCR response, indicating that SHBG effects on OCR were unlikely to be due to changes in mitochondrial capacity (Supplemental Fig. 2b).

**Figure 2:**
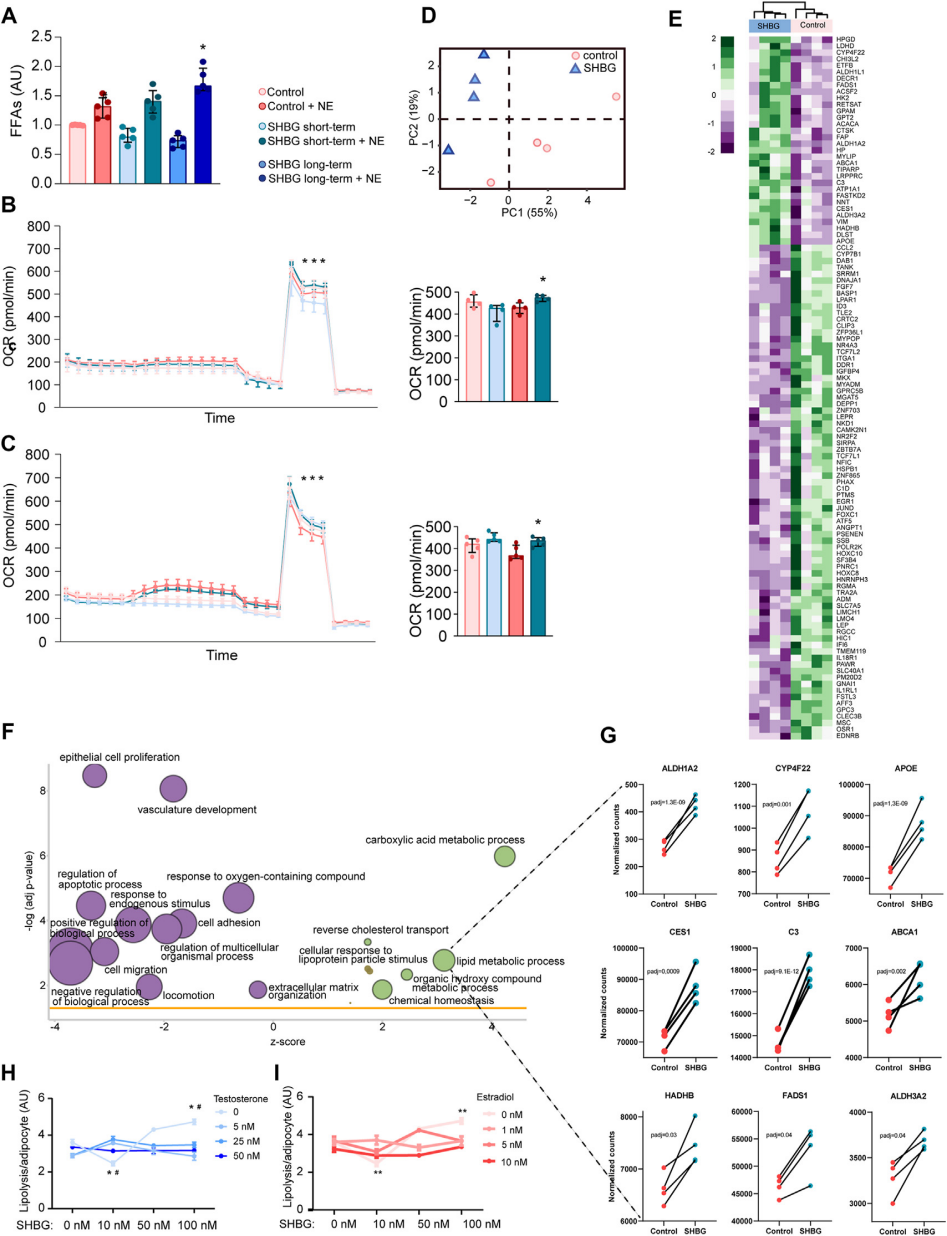
***Effects of sex hormone-binding globulin (SHBG) on lipid metabolism in cultured primary adipocytes***. ***a)*** Basal and nor-epinephrine (NE) stimulated lipolysis in cultured primary subcutaneous white adipocytes stimulated with SHBG or control (short-term = 2 h, long-term = 3 days). ∗ Significantly different from control + NE, p < 0.05. (N = 5). ***b)*** Oxygen consumption rate (OCR) in cultured primary subcutaneous white adipocytes stimulated with short-term SHBG. ∗ Significantly different from control + NE, p < 0.05. Oligomycin (Oligo), Carbonyl cyanide-*p*-trifluoromethoxyphenylhydrazone (FCCP), AntimycinA/Rotenone (A/R). ***c)*** OCR in cultured primary brown adipocytes stimulated with short-term SHBG. ∗ Significantly different from control + NE, p < 0.05. ***d)*** Primary component analysis (PCA) plot of cultured primary subcutaneous white adipocytes stimulated with long-term SHBG vs. control. ***e)*** Heat-map of selected up and down regulated genes related to lipid metabolism in differentiated adipocytes stimulated with long-term SHBG versus control. ***f)*** Gene set enrichment analysis of up- and down regulated pathways based on bulk sequencing of differentiated adipocytes stimulated with long-term SHBG versus control. ***g)*** Most significantly upregulated genes related to lipid metabolic processes, in response to SHBG stimulation. ***h)*** NE-stimulated lipolysis in cultured primary subcutaneous white adipocytes stimulated with long-term SHBG or control in combination with different doses of testosterone (N = 3). ∗ Significantly different from control + NE (0 nM SHBG, 0 nM T), p < 0.05. ***i)*** NE-stimulated lipolysis in cultured primary subcutaneous white adipocytes stimulated with long-term SHBG or control in combination with different doses of estradiol (N = 3). ∗ Significantly different from control + NE (0 nM SHBG, 0 nM E2), p < 0.05. (For interpretation of the references to color in this figure legend, the reader is referred to the Web version of this article.)

To further understand the mechanisms behind the effects of SHBG on adipocyte lipolysis, we performed bulk RNA-seq of long-term SHBG stimulated mature adipocytes. A principal component analysis revealed that adipocytes segregated into two different clusters corresponding to SHBG stimulated and unstimulated adipocytes (Fig 2D). A substantial number of genes related to lipid metabolism were both up- and down regulated in response to SHBG and a gene ontology (GO) analysis further showed that several upregulated pathways were related to lipid metabolism and extracellular matrix remodeling (Figure 2E–F, Supplemental Tables 2–3). Most significantly upregulated genes in SHBG stimulated adipocytes related to lipid metabolic pathways included ALDH1A2, CYP4F22, APOE, CES1, C3, ABCA1, HADHB, FADS1, and ALDH3A2 (Fig 2G). Genes downregulated in SHBG-stimulated adipocytes included LEP, LEPR, FLST3, CRTC2, and ID3, all of which are known to contribute to obesity-induced inflammation and insulin resistance [[40], [41], [42]].

### 3.3 Testosterone and estradiol suppress SHBG induced lipolysis in human adipocytes

SHBG fully liganded to a sex steroid has previously been suggested to cause conformational changes in SHBG, affecting it's ability to bind a potential SHBG receptor. Furthermore, the inhibition of SHBG-receptor binding was proportional with the affinity of the sex steroid binding to the SHBG molecule [21]. Thus, we speculated that the lack of association between SHBG and adipocyte lipolysis in men, as shown in Figure 1, could be a reflection of higher testosterone concentrations (with high SHBG affinity) and lower SHBG concentrations in men compared to women, resulting in significantly less non-steroid bound SHBG molecules. To test this hypothesis, we co-stimulated adipocytes with increasing concentrations of SHBG and testosterone or estradiol. We found that in the absence of sex steroids, NE-stimulated lipolysis increased with increasing concentrations of SHBG. However, the presence of all concentrations of testosterone or estradiol, completely abolished the effects of SHBG on lipolysis. Furthermore, testosterone or estradiol alone had no significant impact on adipocyte lipolytic capacity (Figure 2H and I).

### 3.4 SHBG is taken up by adipocytes upon stimulation and is further endogenously expressed in a small subset of adipocytes

To further explore interactions between SHBG and the adipocyte, we performed SHBG staining following stimulation. Surprisingly, we found that a small subset of the lipid-containing adipocytes stained positive for SHBG even in the absence of supplied SHBG. Upon SHBG stimulation the fraction of adipocytes staining positive for SHBG increased substantially (Figure 3A). 3D visualization of the cells further showed that SHBG accumulated inside the adipocytes indicating that SHBG could be taken up through endocytosis (Figure 3B). To verify that SHBG is internalized by adipocytes, we employed both a non-specific endocytosis inhibitor and siRNA-mediated knockdown of three key regulators of distinct endocytic pathways: Caveolin-1 (CAV1), Dynamin-2 (DNM2), and Megalin (LRP2). Treatment with 2-Deoxy-d-glucose effectively reduced SHBG uptake in 2D images (Figure 3C). However, 3D image analysis revealed a residual SHBG signal (Figure 3D), suggesting that endocytosis was only partially inhibited or that 2-Deoxy-d-glucose generated a background signal. In contrast, siRNA-mediated knockdown of CAV1, DNM2, and LRP2 did not indicate involvement of specific endocytic pathways in SHBG uptake (Supplementary Fig. A4). Although efficient knockdown of DNM2 was achieved, it had no detectable effect on SHBG internalization. Moreover, CAV1 expression remained unchanged following transfection, and LRP2 expression was undetectable in adipocytes.Furthermore, internalization of SHBG was only evident in the lipid-droplet containing adipocytes in the culture. Spatial transcriptomics analysis of human subcutaneous adipose tissue was subsequently performed to investigate potential endogenous SHBG production *in vivo*. However, only a small number of adipocytes displayed a detectable SHBG signal, and this signal was only marginally above background levels (Figure 3E). Several previous studies suggest various subtypes of adipocytes with different phenotypical traits [37,43,44]. We speculated whether a potential SHBG transcription might be localized to any of the previously identified adipocyte subtypes. However, due to the very shallow endogenous expression of SHBG detected with this method, we were unable to attribute the expression to a specific adipose cell subtype. Single nuclei sequencing analyses were used to confirm endogenous expression of SHBG in a small subset (2.6 %) of adipocytes which showed further enrichment in the subcutaneous depot (Figure 3F–G). We did not observe any changes in endogenous SHBG expression in adipocytes upon SHBG stimulation (Supplemental Table 2).

**Figure 3:**
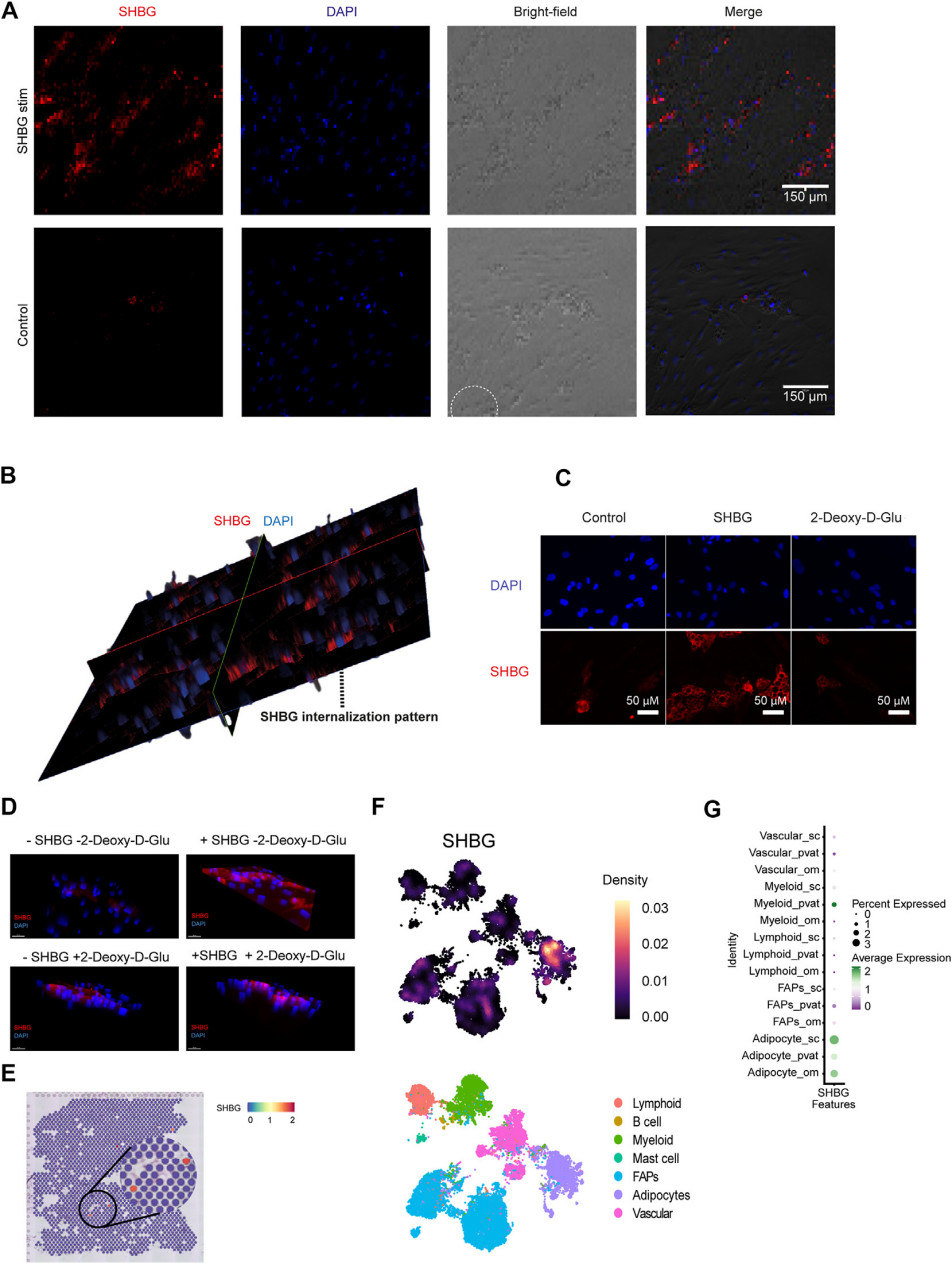
***Sex hormone-binding globulin (SHBG) internalization in cultured primary adipocytes and endogenous SHBG expression in human adipocytes*. *a)*** SHBG protein content in cultured primary subcutaneous white adipocytes stimulated with short-term SHBG vs. controls. ***b)*** 3D-model of SHBG content in differentiated cultured adipocytes stimulated with short-term SHBG. ***c)*** Inhibition of SHBG Endocytosis in Human Adipocytes by 2-Deoxy-d-Glucose and untreated controls (scale bar = 50 μM) ***d)*** 3D representation of SHBG endocytosis in human adipocytes: inhibition by 2-Deoxy-d-Glucose compared to controls (scale bar = 50 μM) ***e)*** Endogenous SHBG expression in human adipocyte subtypes assessed through spatial transcriptomics. ***f)*** Endogenous SHBG expression specific for the adipocyte subpopulation of cellular subtypes assessed through single nuclei RNA sequencing (snRNAseq). ***g)*** Endogenous SHBG expression in adipose subpopulation from differing adipose depots (sub cutaneous (sc), perivascular (pvat), and omental (om)) based on snRNAseq.

## 4 Discussion

In the current study, we investigated the possibilities for direct effects of SHBG, in the presence and absence of sex hormones, on adipocyte lipolytic function. We demonstrate that, circulating SHBG might play a direct regulatory role in adipocyte function in women only, where it promotes stimulated lipolysis in combination with lower basal lipolysis, both associated with improved whole-body insulin sensitivity. Contrary, the lipolytic phenotype associated with low SHBG, manifested as low catecholamine-induced lipolysis despite high levels of basal lipolysis, reflects metabolic inflexibility and is a key determinant of impairments in adipocyte metabolic health and has been shown to predict weight gain and insulin resistance over time [22,39,45,46]. However, no associations between serum SHBG and adipocyte lipolysis were detected in a cohort of men. The sex dependent differences in the relationship between SHBG and adipocyte lipolytic capacity indicate that metabolic benefits from high serum SHBG might vary across sexes. This is in accordance with several previous studies, where the relationship between low serum SHBG and increased risk of lipid metabolic disease and type 2 diabetes were more pronounced in women compared to men [12,14,15]. Studies suggest that testosterone binding to SHBG leads to conformational changes in the SHBG molecule, which could prevent SHBG from binding to its receptor [21,47]. As only 18 % of SHBG binding sites are occupied by steroids in women versus 56 % in men (with some individual variation depending on serum SHBG and sex hormone concentrations), this model provides a reasonable explanation for why we only observed adipocyte lipolytic effects of SHBG in women and in the absence of testosterone, *in vitro* [48].

The presence and function of a potential SHBG receptor has been widely debated over the last decades and previous studies have suggested signaling through both G-protein coupled receptors and endocytosis through Megalin [6,19,21,49]. However, the characteristics and identity of an SHBG receptor in humans remains to be solidified. To investigate whether the communication between adipocytes and the SHBG molecule was mediated through direct binding of SHBG to the adipocyte, we performed immunoflouresence analyses on SHBG stimulated adipocytes, *in vitro*. We found that SHBG accumulated intracellularly following SHBG stimulation in the lipid containing adipocytes. These findings support the hypothesis of an endocytic uptake of SHBG, at least in adipocytes. However, whether the lipolytic effects of SHBG depends on preceding endocytosis remains to be clarified. LDL receptor related protein 2 (LRP2), the gene encoding for Megalin, could be a potential candidate to mediate this endocytosis. However, LRP2 is only lowly expressed in mature adipocytes.

Surprisingly, immunofluorescence microscopy demonstrated that some lipid-containing adipocytes stained positive for SHBG, even without stimulation with purified SHBG. Through transcriptome analyses, an endogenous production of SHBG in a small subset of adipocytes was confirmed. However, deeper transcriptomic characterization of this population of adipocytes was not possible due to the low overall abundance. Even though the main production of circulating SHBG is derived from the liver, several other organs have been shown to produce lower concentrations of SHBG endogenously, including the brain, kidney, prostate, uterus, and testes [[50], [51], [52], [53]]. The role of extrahepatic SHBG production is largely unknown and the secretory potential debated. However, both endogenous accumulation and paracrine secretion has been suggested as means to stabilize or potentiate local tissue-specific concentrations of sex hormones [54].

We performed global transcriptome analyses on SHBG-stimulated versus unstimulated adipocytes and found that adipocytes increase the expression of several pathways related to lipid metabolism upon stimulation with SHBG. These experiments clearly demonstrated that SHBG itself acts as a regulator of adipocyte metabolism, activating a transcriptional program that controls lipid metabolism upon *in vitro* stimulation.

[55,56] We also observed an upregulation of extracellular matrix processes upon SHBG stimulation. Interestingly, substantial extracellular sequestration of SHBG has previously been observed in stromal tissues, through SHBG-binding of the ECM-associated protein fibulin, which, in our hands, was among the upregulated genes in adipocytes upon SHBG stimulation [57]. It is therefore reasonable to speculate that SHBG-induced conformational changes in the extracellular environment may also enhance local SHBG signaling within adipose tissue.

This study describes, in part, how SHBG regulate key metabolic processes in adipocytes. Several studies further suggest a lipid-dependent regulation of hepatic SHBG synthesis indicating a liver-adipose metabolic axis. Thus, hepatic *de novo* lipogenesis has been shown to inhibit SHBG synthesis through inhibition of HNF4-α [58] explaining the close relationship between increased intrahepatic lipid accumulation and decreased circulating SHBG. However, this relationship is only applicable in women and not men [59]. Furthermore, adiponectin has been suggested to stimulate SHBG production in the liver through activation of AMPK [60].

The study has some limitations. While we compared adipocyte lipolytic capacity across sexes in the human cohorts, there were fundamental phenotypic differences between the sexes which might impact our results. Thus, men were significantly younger and had lower BMIs compared to the women. Furthermore, the cohort of men were considerably smaller with less variation in circulating SHBG. We performed sensitivity analyses to assess if the lack of associations between circulating SHBG and adipocyte lipolytic capacity in men could be attributed to outliers within the dataset, which was not the case. Furthermore, power analyses were performed to investigate if the findings could be explained from lack of power. However, future studies should aim to replicate these experiments in bigger cohorts.

In conclusion, we here suggest a novel pathway of liver to adipose crosstalk through SHBG. We find that SHBG improves lipolytic function of the adipocytes as well as upregulates several lipid metabolic pathways, but only in the absence of testosterone. These findings might explain why adipocyte lipolysis in mature adipocytes were only associated with circulating SHBG in women. Overall, the study contributes to the understanding of the underlying molecular mechanisms behind the beneficial metabolic effects of SHBG.

## CRediT authorship contribution statement

**Julie Abildgaard:** Writing – original draft, Project administration, Methodology, Investigation, Formal analysis, Data curation, Conceptualization. **Aiste Aleliunaite:** Writing – review & editing, Methodology, Investigation. **Carla Horvath:** Writing – review & editing, Methodology, Investigation. **Nagendra Palani:** Writing – review & editing, Visualization, Formal analysis, Data curation. **Tora Ida Henriksen:** Writing – review & editing, Methodology, Investigation. **Jiawei Zhong:** Writing – review & editing, Visualization, Investigation, Formal analysis. **Katja Munch Lorentsen:** Data curation, Writing – review & editing. **Victor Svenstrup:** Writing – review & editing, Methodology, Investigation. **Hanne Frederiksen:** Writing – review & editing, Investigation. **Anders Juul:** Writing – review & editing, Investigation. **Camilla Charlotte Scheele:** Writing – review & editing, Methodology, Investigation. **Søren Nielsen:** Writing – review & editing, Writing – original draft, Supervision, Project administration, Methodology, Investigation, Formal analysis, Data curation, Conceptualization.

## Declaration of competing interest

The authors declare that they have no conflicts of interest to disclose in relation to this manuscript. No financial or personal relationships with organizations or individuals could have influenced the content or conclusions of this work.

## Figures and Tables

**figs1:**
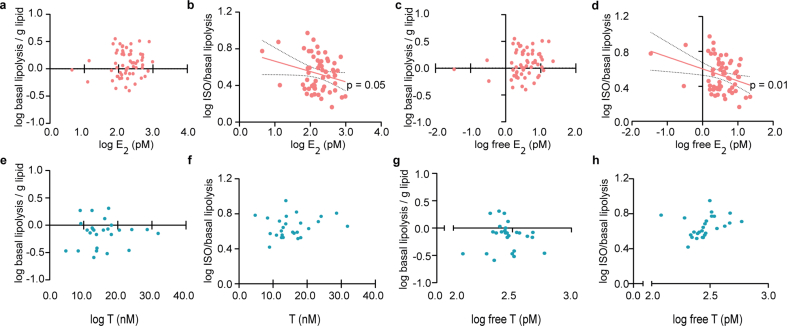


**figs2:**
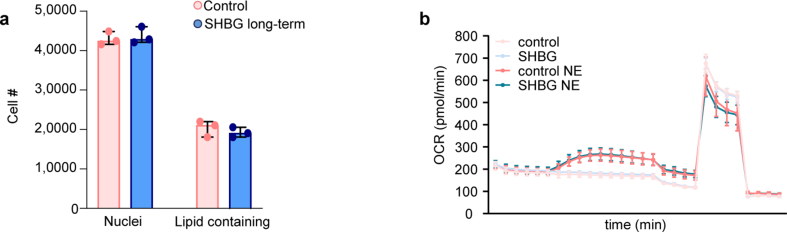


**figs3:**
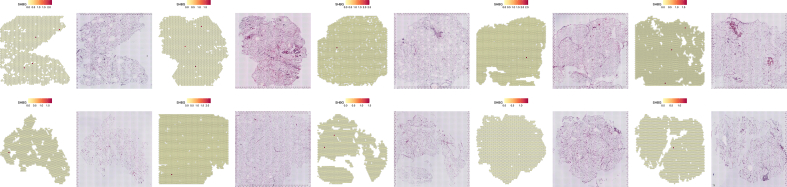


**figs4:**
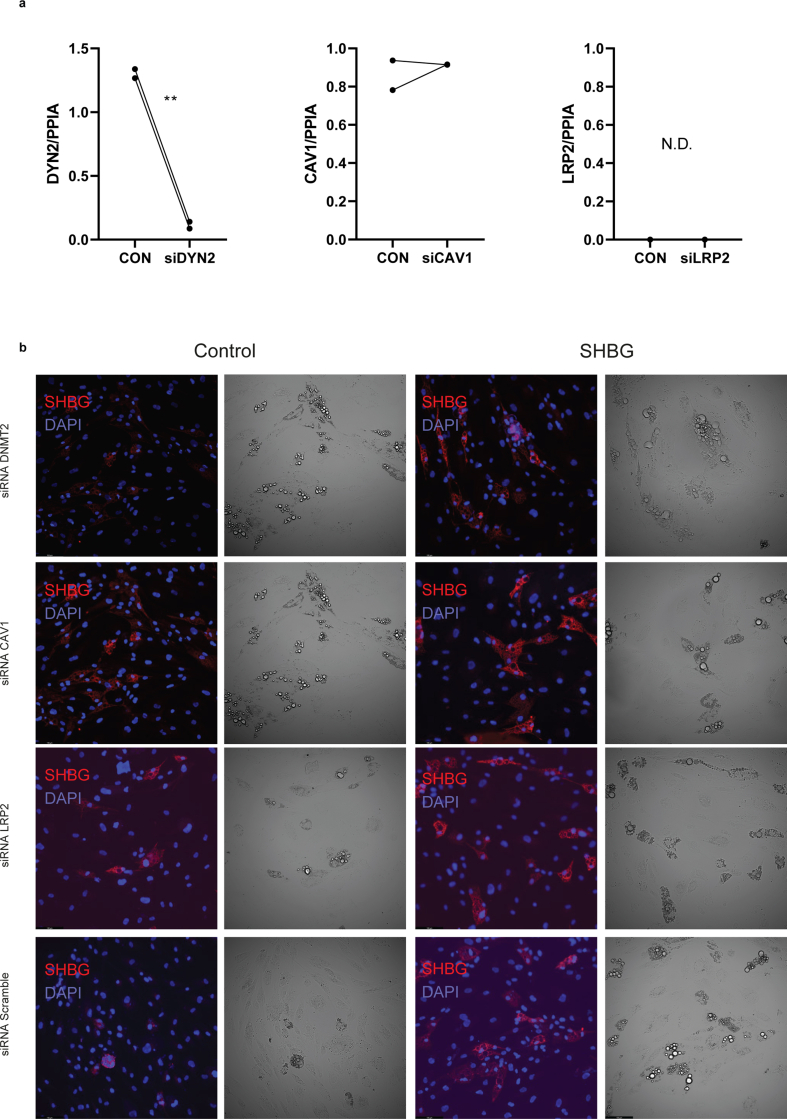


## Data Availability

Data will be made available on request.
